# Anterior cleft palate due to Cbfb deficiency and its rescue by folic acid

**DOI:** 10.1242/dmm.038851

**Published:** 2019-06-27

**Authors:** Safiye E. Sarper, Toshihiro Inubushi, Hiroshi Kurosaka, Hitomi Ono Minagi, Yuka Murata, Koh-ichi Kuremoto, Takayoshi Sakai, Ichiro Taniuchi, Takashi Yamashiro

**Affiliations:** 1 Department of Orthodontics and Dentofacial Orthopedics, Graduate School of Dentistry, Osaka University, Osaka 565-0871, Japan; 2 Laboratory of Theoretical Biology, Graduate School of Sciences, Osaka University, Osaka 565-0871, Japan; 3 Department of Oral-facial Disorders, Osaka University Graduate School of Dentistry, Osaka 565-0871, Japan; 4 Department of Advanced Prosthodontics, Graduate School of Biomedical & Health Sciences, Hiroshima University, Hiroshima 734-8553, Japan; 5 Laboratory for Transcriptional Regulation, RIKEN Research Center for Allergy and Immunology, Yokohama 230-0045, Japan

**Keywords:** Stat3, Palatogenesis, Palatal fusion, Epithelial disintegration, Runx transcription factors, Tgfb

## Abstract

Core binding factor β (Cbfb) is a cofactor of the Runx family of transcription factors. Among these transcription factors, Runx1 is a prerequisite for anterior-specific palatal fusion. It was previously unclear, however, whether Cbfb served as a modulator or as an obligatory factor in the Runx signaling process that regulates palatogenesis. Here, we report that Cbfb is essential and indispensable in mouse anterior palatogenesis. Palatal fusion in *Cbfb* mutants is disrupted owing to failed disintegration of the fusing epithelium specifically at the anterior portion, as observed in *Runx1* mutants. In these mutants, expression of TGFB3 is disrupted in the area of failed palatal fusion, in which phosphorylation of Stat3 is also affected. TGFB3 protein has been shown to rescue palatal fusion *in vitro*. TGFB3 also activated Stat3 phosphorylation. Strikingly, the anterior cleft palate in *Cbfb* mutants is further rescued by pharmaceutical application of folic acid, which activates suppressed Stat3 phosphorylation and *Tgfb3* expression *in vitro*. With these findings, we provide the first evidence that Cbfb is a prerequisite for anterior palatogenesis and acts as an obligatory cofactor in the Runx1/Cbfb-Stat3-Tgfb3 signaling axis. Furthermore, the rescue of the mutant cleft palate using folic acid might highlight potential therapeutic targets aimed at Stat3 modification for the prevention and pharmaceutical intervention of cleft palate.

## Abstract (precis)

**Summary:** Epithelial deletion of Cbfb results in an anterior cleft palate with impaired fusion of the palatal process; folic acid application rescues the mutant phenotype with Stat3 activation *in vitro*.

## INTRODUCTION

Cleft palate is one of the most common congenital anomalies in humans and its etiology is complex (Dixon et al., 2011; Murray, 2002). The palate is derived from the primary and secondary palates, which are located in the anterior and posterior portions of the palate, respectively (Gu et al., 2008). Palatal fusion is essential in palatogenesis and defects in the fusion process lead to cleft palate. Two halves of the palatal process fuse in the middle to form the secondary palate, which further fuses with the primary palate and the nasal septum to form the definite palate (Ferguson, 1988).

Among various molecules regulating palatogenesis, Runt-related transcription factor 1 (Runx1) is involved in the regulation of palatal fusion specifically in the anterior region. Epithelial-specific loss of *Runx1* results in failure of palatal fusion, specifically at the anterior portion between the primary and the secondary palate, with failed disintegration of the medial-edge epithelium. In these mutants, the expression of transforming growth factor β (*Tgfb3*), a crucial regulator of palatal fusion, is disrupted among various molecules regulating palatogenesis, and the application of TGFB3 protein is able to rescue the mutant cleft palate; thus, Tgfb3 is crucial for Runx1 signaling in palatogenesis (Sarper et al., 2018). In the Runx mutants, Stat3 phosphorylation was downregulated specifically at the anterior palate. Moreover, a Stat3 inhibitor disrupted anterior palatal fusion and this was accompanied by downregulation of *Tgfb3* expression *in vitro.* These observations indicate that the Runx1-Tgfb3 signaling axis is mediated by Stat3 phosphorylation (Sarper et al., 2018). These findings also suggest that extrinsic modification of Stat3 activity affects Tgfb3 signaling, and might be a potential therapeutic target in pharmaceutical intervention for cleft palate (Sarper et al., 2018).

Core binding factor β (Cbfb) is a cofactor of the Runx family of transcription factors (Runx1, Runx2 and Runx3); Runx proteins form a heterodimeric transcription complex with Cbfb (Huang et al., 2001). Cbfb enhances the binding affinity of the complex for DNA and promotes Runx protein stability (Huang et al., 2001; Ogawa et al., 1993; Wang et al., 1993). Of note, Cbfb can act as either an obligate cofactor for the Runx function or as a dispensable modulator of Runx activity (Gau et al., 2017). For example, Cbfb acts as an obligate cofactor for the Runx function in hematopoietic cells (Chen et al., 2011), but as a dispensable modulator of Runx activity in skeletogenesis (Yoshida et al., 2002). The possible functional role of Cbfb in palatogenesis has not been investigated, however.

A human genome study demonstrated that *CBFb* haploinsufficiency owing to an interstitial deletion caused cleft palate and congenital heart anomalies in humans (Khan et al., 2006; Tsoutsou et al., 2013; Yamamoto et al., 2008). A chromosomal fragile site of FRA16B, which colocalizes with breakpoints within *CBFb* at the chromosomal locus 16q22.1., is also involved in the inheritance of cleft palate (McKenzie et al., 2002). However, whether Cbfb is an obligate cofactor or a dispensable modulator in Runx1 signaling in palatogenesis has not been investigated.

Maternal folic acid supplementation has been shown to be an effective intervention for reducing the risk of non-syndromic cleft palate (Millacura et al., 2017; Wehby and Murray, 2010); however, the mechanism by which folic acid prevents such structural anomalies in the fetus is still unknown (Obican et al., 2010). Interestingly, folic acid and folate can activate Stat3 (Hansen et al., 2015; Wei et al., 2017). Our previous study showed that pharmaceutical application of Stat3 inhibitors disrupts palatal fusion with downregulation of *Tgfb3*. Hence, it was assumed that folic acid might be a useful therapy for preventing cleft palate through the extrinsic modification of Stat3 activation.

Here, we report the first evidence that Cbfb is essential in anterior palatogenesis as an obligatory cofactor in the Runx1/Cbfb-Stat3-Tgfb3 signaling axis. In addition, we demonstrate the rescue of mutant cleft palate through pharmaceutical folic acid application, at least in part, by activating Stat3 phosphorylation in the Runx1/Cbfb-Stat3-Tgfb3 signaling axis during palatogenesis.

## RESULTS

### Palatal phenotypes in Cbfb mutants

The palatal phenotype was evaluated *in vivo* to see how Cbfb affects palatal fusion using epithelial-specific conditional knockout (*K14-Cre/Cbfb^fl/fl^*) mice (Kurosaka et al., 2011). The recombination efficiency of K14-Cre was evaluated in the developing palate previously (Sarper et al., 2018); this earlier study also demonstrated that K14 was expressed in the palatal epithelium in the embryonic stage.

**Fig. 1.**
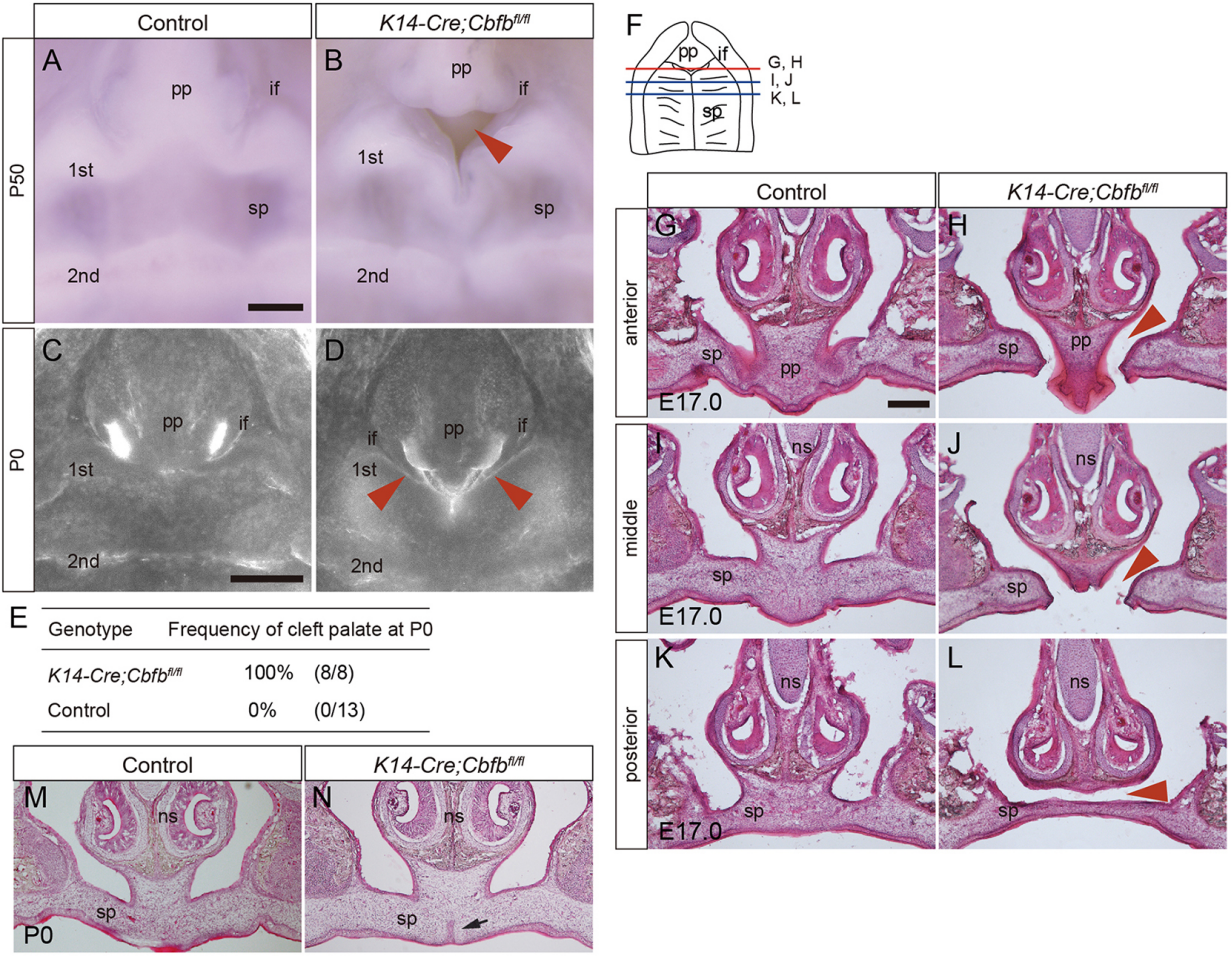
**Palatal phenotypes of *K14-Cre/Cbfb^fl/fl^* mice.** (A-D) Occlusal views of control and *Cbfb* mutant mouse palates by direct observation through a dissecting microscope (A,B) or by confocal projection of DAPI-stained samples (C,D). An anterior cleft palate was evident at the boundary between the primary and secondary palates in *Cbfb* mutant palates both at P50 (A,B) and P0 (C,D). The arrowheads indicate the cleft. (E) The frequency of anterior cleft in control and *Cbfb* mutant mice at P0. (F) Diagram showing the occlusal view of the palate and the section positions, as indicated by the lines. (G-L) Histological sections at E17.0 revealed that the palatal shelves of *Cbfb* mutant mice did not make contact at the boundary between the primary and secondary palate (G,H,J,K). In the more posterior region, the secondary palate was fused completely;however, the fused palate did not make contact with the inferior border of the nasal septum (I,L). Arrowheads indicate the failure of fusion. (M,N) Histological sections at P0 revealed that the epithelial remnants were retained at the anterior-most part of the secondary palate in the *Cbfb* mutant mice (arrow). Epithelial remnants between the nasal process and secondary palate were completely removed at the interface in both the controls and mutants. 1st, first palatine rugae; 2nd, second palatine rugae; if, incisive foramen; ns, nasal septum; pp, primary palate; sp, secondary palate. Scale bars: 1000 μm in A (P50); 400 μm in A (P0); 200 μm in G-N.

In the *Cbfb* mutants, anterior cleft was evident between the primary and secondary palates both at postnatal day (P)0 and P50 (Fig. 1A-D) by direct observation through a dissecting microscope or by confocal projection of DAPI-stained samples. The cleft was seen in 100% of the mutants (*n*=8) when evaluated at P0 (Fig. 1E). The anterior cleft in the P50 mice became larger in comparison with the P0 mice. It is likely that the anterior-posterior dimension of the maxillary complex became larger owing to postnatal growth. In histological sections (Fig. 1F), failed palatal fusion was also confirmed at the first rugae anterior to posterior (AP) level in *Cbfb* mutants at embryonic day (E)17.0 (Fig. 1G-J). In the more posterior portion, the secondary palate did not make contact with the primary palate or the nasal septum (Fig. 1K,L). At this stage, the distance between the unfused palatal process at the interface between the primary palate and the nasal septum was 306±29.9 µm at the second rugae level (mean±s.d.; arrowhead, Fig. 1L). In the mutants, the secondary palate exhibited a partial submucous cleft with retained epithelial remnants at the anterior-most region of the secondary palate at P0 (Fig. 1M,N).

To define the moment of cleft origin and the maintenance of the phenotypes, we further evaluated sequential histological sections from E14.0 to E16.0 (Figs S1, S2). At E14.0, the palatal processes were apart, and no fusion among the processes was evident (Fig. S1A-G). At E14.5, the secondary palatal process started to fuse at the middle of the palate, although the secondary palate had not made contact with the primary palate (Fig. S1J,K,M,N). By E14.5, palatal phenotypes of the *Cbfb* mutant were not evident on the series of histological sections (Fig. S1). At E15.0, the epithelial remnants at the midline of the secondary palates start to be degraded and mesenchymal confluence was partially achieved in both the control and mutant mice at the 2nd rugae level (Fig. S2D,D′,G,G′). The epithelial remnants were also evident at the interface between the primary and the secondary palates in the control (Fig. S2D,D′). By contrast, some of the mutants at E15.0 had contact between the primary and the secondary palates without any mesenchymal confluency (Fig. S2F-G′) and some had no contact, as observed in *Runx1* mutants. At E16.0, degrading epithelium was still evident at the anterior-most portion of the interface between the secondary palatal process in both the control and mutant mice (Fig. S2I,I′,L,L′). The degrading epithelium was still evident at the interface between the primary and secondary palates in control mice (Fig. S2J,J′). At E16.0, *Cbfb* mutants had no contact between the primary and the secondary palates (Fig. S2K-L,K′-L′). It is likely that the primary palatal process that made contact at E15.0 became detached by E16.0. Collectively, the morphological differences in *Cbfb* mutants were not evident by E14.5; however, the epithelial fusion between the primary and secondary palates started to be disrupted around E15.0 and the palatal phenotypes became evident from E16.0.

### Characterization of the mutant epithelium in palatal fusion

In palatal fusion, the medial-edge epithelium terminates to proliferate and enters apoptosis (Cuervo and Covarrubias, 2004; Cui et al., 2005). The periderms covering the fusing epithelium are sloughed off (Hu et al., 2015). The intervening epithelium then needs to be degraded in order to achieve mesenchymal confluence (Gritli-Linde, 2007). Our previous study using *K14-Cre;R26R* mice demonstrated intense β-gal-positivity (an indicator for Cre activity) among the cells at the epithelium overlying the palatal process of the secondary and primary palates (Sarper et al., 2018). This was also evident in the contacting and fused epithelium in anterior palatogenesis.

**Fig. 2.**
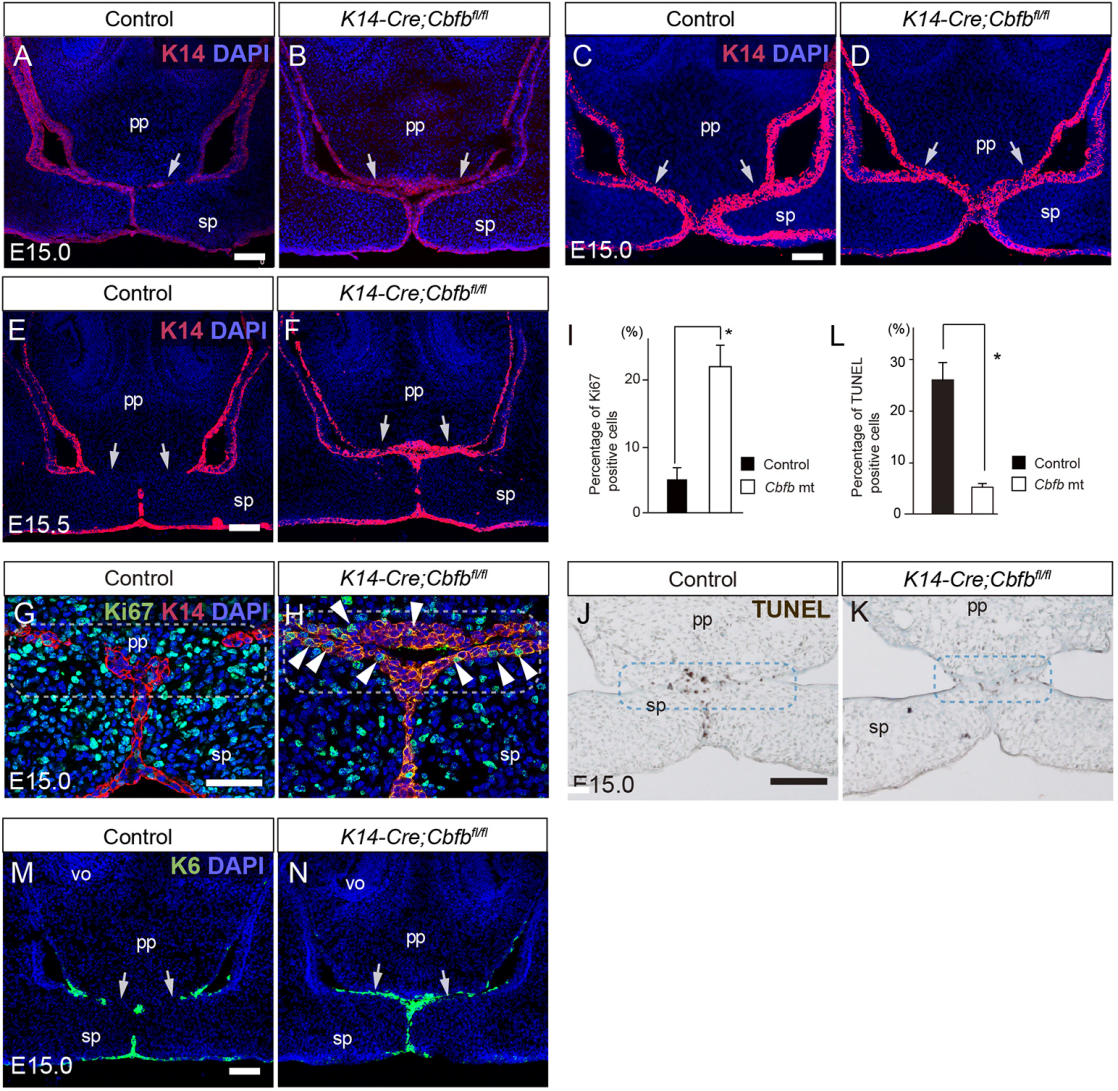
**Palatal phenotypes in *Cbfb* mutant mice.** (A-F) Immunostaining for K14 during anterior palatogenesis at E15.0 (A-D) and at E15.5 (E,F). (A) In controls, K14-labeled epithelial seams were formed at the boundary between the primary and secondary palate at E15.0 (arrows). (B) In *Cbfb* mutants, K14-labeled epithelial remnants were retained on the contacting palatal shelves (arrows). (C,D) At the anterior-most regions, K14-labeled epithelial remnants were retained in both controls and mutants (arrows). (E,F) At E15.5, the epithelial remnants between the primary and secondary palates (arrows) were resolved in the control (E) and retained in the mutants (F). (G,H) Double immunohistochemical staining for Ki67 (green) and K14 (red) showed that Ki67 signals were sparse in the epithelial remnants in the wild-type palates (G), whereas some Ki67-positive epithelium (arrowheads) were retained in *Cbfb* mutants at the interface between the primary and secondary palate (H). Dashed frame defines area that was quantified in I,L. (I) There were significantly more Ki67-positive epithelial cells in the *Cbfb* mutants than in the wild-type palates. (J,K) TUNEL-positive cells were evident in the epithelial remnants localized at the boundary between the primary and the secondary palate in wild type (J), whereas there were fewer TUNEL-positive cells in the epithelial remnants in *Cbfb* mutants (K). (L) The percentage of TUNEL-positive cells at the interface between the primary and secondary palate (dashed frame) was significantly lower in the *Cbfb* mutants. (M,N) Immunohistochemical staining for K6 (green) revealed that K6-positive periderms were retained on the unfused epithelial surface of the nasal side of the secondary palate and the nasal septum in the *Cbfb* mutants. The nuclei were counterstained with DAPI (blue). The arrows indicate persistent periderm. ns, nasal septum; pp, primary palate; sp, secondary palate; vo, vomeronasal organ. *P<0.05 (Mann-Whitney *U*-test; data are mean+s.d.). Scale bar: 100 μm in A-F,M,N; 50 μm in G-H; 200 μm in J,K.

At E15.0, immunostaining of K14 revealed that the epithelium seam was present sparsely at the boundary between the primary and secondary palates in the control, whereas there was partial contact but no fusion in the mutant palatal epithelium between the primary and secondary palates (Fig. 2A,B) and in the anterior-most region of the secondary palate (Fig. 2C,D).

At E15.5, K14-immunostained cells in control palates exhibited further degradation of the epithelial remnants at the interface between the primary and secondary palates, whereas the epithelium was retained in the mutant primary and secondary palates (Fig. 2E,F).

The proliferative activity was evaluated using Ki67 staining at the interface between the primary and secondary palates. Double-staining for Ki67 and K14 immunoreactivity showed that Ki67-positive proliferating cells were sparse at the fused epithelium in wild type (Fig. 2G), whereas some immunoreactivity was retained in the contacting epithelium in *Cbfb* mutants (Fig. 2H). Ki67-positive cells at the interface between the primary and secondary palate were quantified from the images and we found that Ki67/K14-double-positive proliferating epithelial cells were retained more in the contacting epithelium of the *Cbfb* mutant mice than in the fusing epithelium of the control mice (Fig. 2I).

TUNEL assay showed that apoptotic signals were evident in the fused epithelium in controls (Fig. 2J), whereas far fewer signals were detected in the unfused epithelium of the *Cbfb* mutants (Fig. 2K). TUNEL-positive cells at the interface between the primary and secondary palate were quantified from the images. We found the percentage of TUNEL-positive cells at the fusing epithelium to be significantly reduced in the mutants compared with controls (Fig. 2L).

During palatogenesis, the periderm of the secondary palate transiently covers the fusing palatal process and is sloughed before palatal fusion (Hu et al., 2015). Keratin 6 (K6) detects periderm (Richardson et al., 2014) and K6 immunoreactivity was sparsely observed in the epithelial remnants in the anterior regions of E15.0 wild-type mice (Fig. 2M). By contrast, K6-immunoreactive periderms in *Cbfb* mutants were retained on the unfused epithelial surface of the primary palate, on the nasal side of the secondary palate and on the nasal septum, indicating that the periderm had not been sloughed off at the anterior region of the palate as a consequence of *Cbfb* deficiency (Fig. 2N).

Taken together, these findings show that *Cbfb* is essential for anterior palatal fusion and suggest that defective palatal fusion in *Cbfb* mutants could result from the failed disintegration of the epithelium in the anterior palate, as observed in *Runx1* mutants (Sarper et al., 2018).

### The expression of Cbfb mRNA in the developing palate

**Fig. 3.**
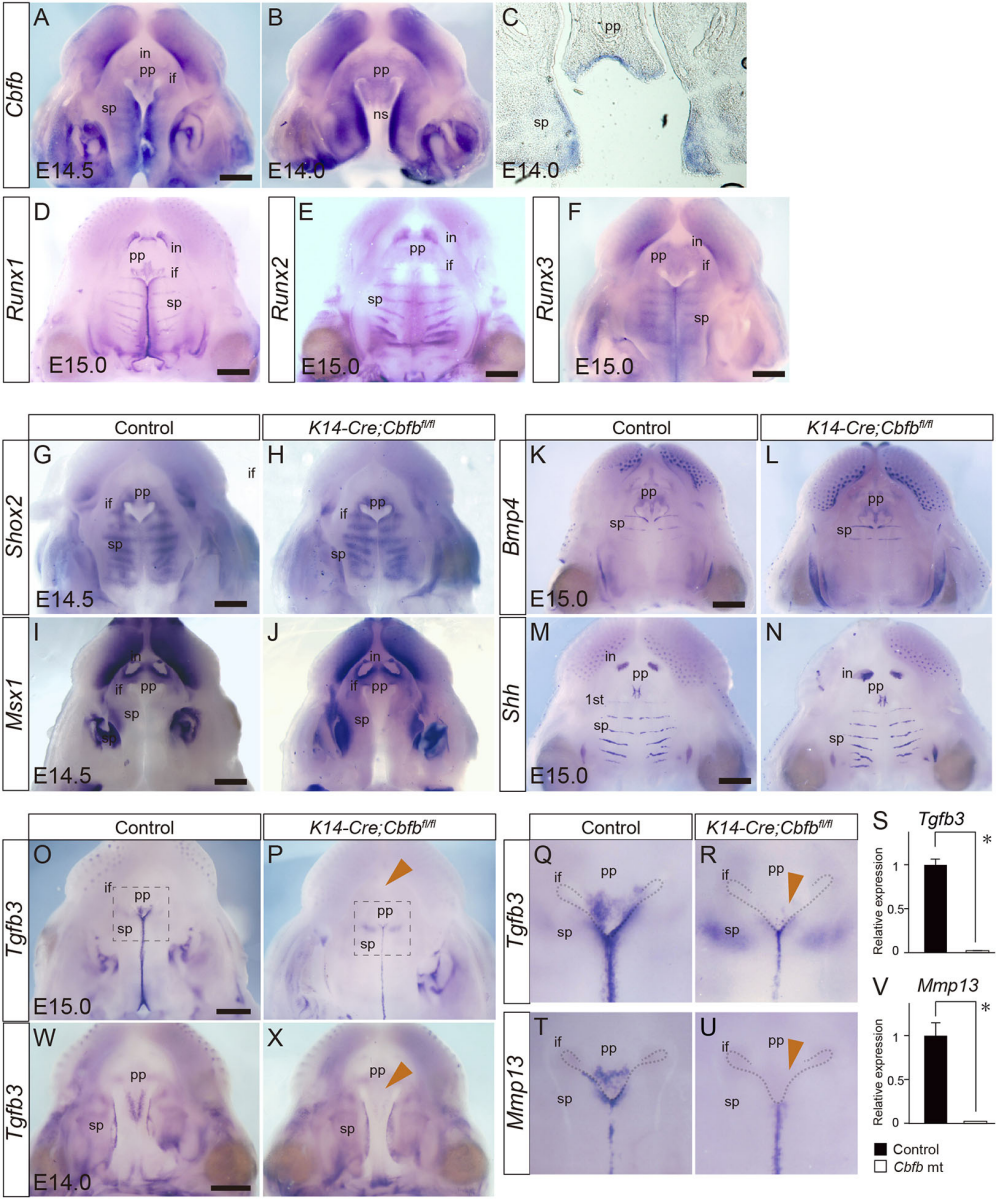
**Gene expression during palatogenesis in *Cbfb* mutants.** (A-F) Expression of *Cbfb*, *Runx1*, *Runx2* and *Runx3* in the developing palate of the wild type. *Cbfb* was widely distributed along the AP axis and not specifically in the anterior regions, as shown by whole-mount *in situ* hybridization (A,B). *Cbfb* was expressed both in the epithelium and the mesenchyme (C). Whole-mount *in situ* hybridization of *Runx1* (D), *Runx2* (E) and *Runx3* (F) mRNA in the developing palate of wild-type mice. (G-N) Whole-mount *in situ* hybridization of *Shox2* (G,H), *Msx1* (I,J), *Bmp4* (K,L) and *Shh* (M,N) mRNA in the developing palate of *Cbfb* mutant and wild-type mice. The *Shox2*, *Msx1*, *Shh* and *Bmp4* expression was not altered by *Cbfb* deficiency. (O,P) Whole-mount *in situ* hybridization of *Tgfb3*. The *Tgfb3* expression was markedly downregulated at the fusing epithelium at the primary palate and at the anterior-most portion of the secondary palate in *Cbfb* mutant mice (arrowhead). (Q,R,T,U) Higher magnification of boxed areas in O and P whole-mount images of the *Tgfb3* and *Mmp13*. The expression of both *Tgfb3* and *Mmp13* was markedly affected in *Cbfb* mutants (arrowheads). (S,V) qPCR analysis confirmed the remarkable downregulation of *Tgfb3* (S) and *Mmp13* (V) in *Cbfb* mutants. (W,X) Whole-mount *in situ* hybridization of *Tgfb3* at E14.0. Downregulated expression of *Tgfb3* was evident at the primary palate in *Cbfb* mutant mice (arrowhead). if, incisive foramen; in, incisal tooth bud; ns, nasal septum; pp, primary palate; sp, secondary palate. *P<0.05 (Mann-Whitney *U*-test; data are mean+s.d.). Scale bars: 500 μm.

The whole-mount *in situ* hybridization showed that *Cbfb* transcripts were widely distributed along the AP axis and not specifically in the anterior regions at E14.0 (Fig. 3A,B). The distribution of the *Cbfb* mRNA expression, therefore, does not explain why *Cbfb* deficiency caused an anterior-specific phenotype in palatogenesis. Sliced sections revealed that *Cbfb* transcripts were present in both the palatal epithelium and mesenchymal tissue (Fig. 3C). *Runx1* expression was intense in the fusing region of the palatal shelves and in the primary palate regions (Fig. 3D) and *Runx2* expression was present in the fusing region of the palatal process; however, *Runx2* expression was lower in the primary palate region than in the secondary palate (Fig. 3E), as previously reported (Charoenchaikorn et al., 2009). *Runx3* was also detected in the fusing region of the palatal process (Fig. 3F).

### Altered mRNA expression in the Cbfb mutant palate

To clarify the molecular mechanisms underlying the failed palatal fusion in *Cbfb* mutants, we evaluated the changes in several molecules that have been recognized as anterior-specific genes in palatogenesis. Whole-mount *in situ* hybridization revealed that the distribution of *Shox2*, *Msx2*, *Bmp4* and *Shh* (Baek et al., 2011; Hilliard et al., 2005; Li and Ding, 2007; Welsh and O'Brien, 2009) expression was not altered by *Cbfb* deficiency (Fig. 3G-N). However, expression of *Tgfb3* was significantly decreased in *Cbfb* mutants in the anterior region of the palate (Fig. 3O,P). Higher magnification views demonstrated that significant decreases in *Tgfb3* signals were evident in the primary palate regions (Fig. 3Q,R). Quantitative real-time PCR (qPCR) analysis of the microdissected tissue also showed the downregulation of *Tgfb3* in the primary palate (Fig. 3S). *Mmp13* lies downstream of *Tgfb3* signaling in palatogenesis (Blavier et al., 2001). A higher magnification view of *Mmp13* expression also demonstrated a significant decrease in signals in the primary palate regions and at the anterior-most secondary palate corresponding to the first and second rugae (Fig. 3T,U). qPCR analysis of microdissected tissue confirmed marked downregulation of *Mmp13* expression in the primary palate (Fig. 3V). Downregulated Tgfb3 expression in the anterior region of the mutant palate was also evident at E14.0 (Fig. 2W,X). These findings indicate that *Tgfb3* is a key target in *Cbfb* mutants and demonstrate that the *Shh*, *Shox2* and *Msx1-Bmp4* pathways are not affected, as observed in *Runx1* mutants (Sarper et al., 2018).

### Rescue of cleft palate in Cbfb mutant mice by TGFB3

Given the crucial roles of Tgfb3 in palatogenesis, downregulation of *Tgfb3* expression in *Cbfb* mutants might account for the failure of palatal fusion. Therefore, we further investigated whether TGFB3 protein can rescue the cleft palate in *Cbfb* mutants.

**Fig. 4.**
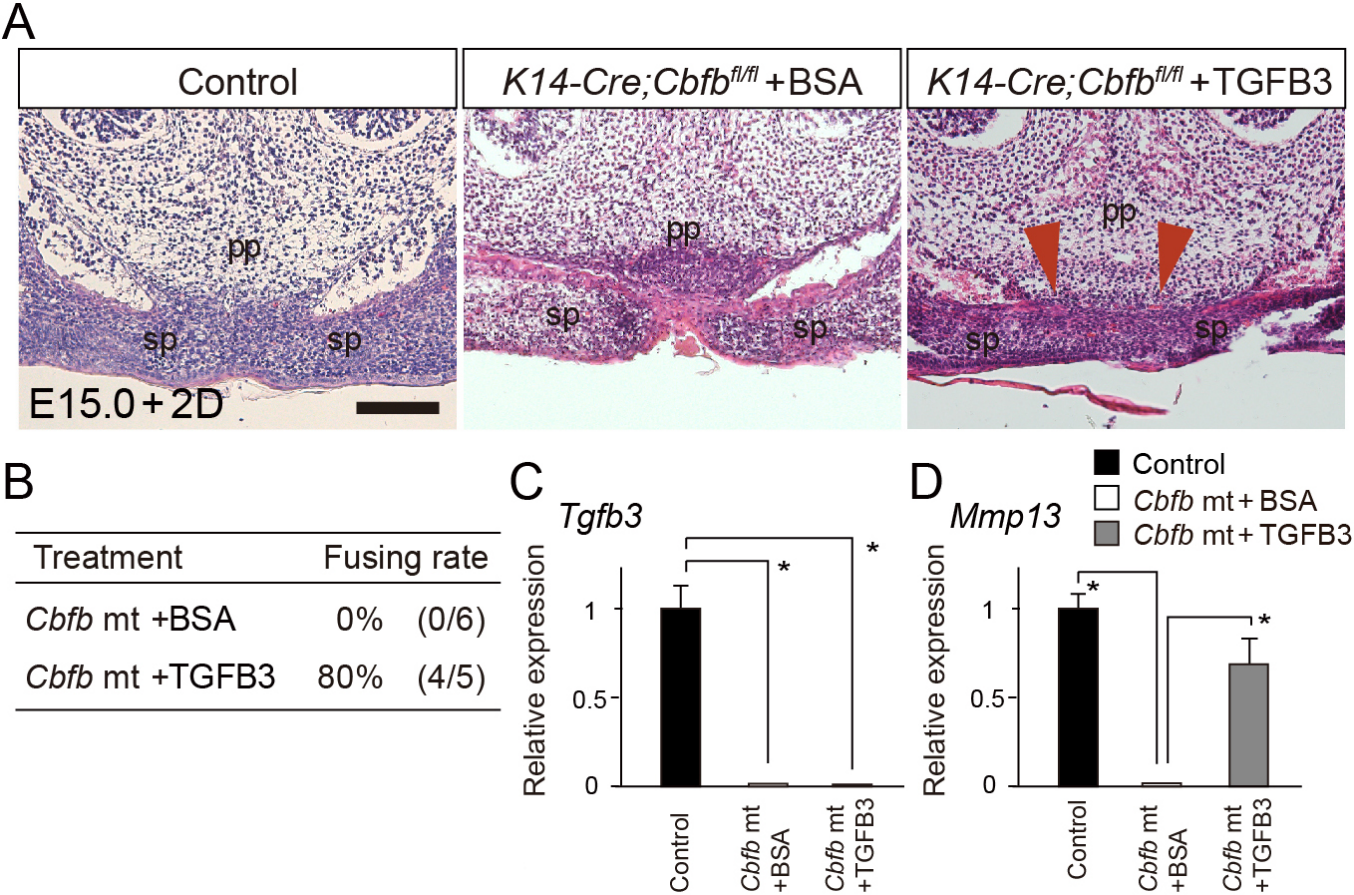
**TGFB3 rescues cleft palate of *Cbfb* mutants.** (A) Histological sections showed that failure of the palatal fusion in *Cbfb* mutants was partially rescued by TGFB3 protein beads in culture (arrowheads). (B) The rescue ratio of the cleft palate in *Cbfb* mutants by TGFB3 application. (C,D) qPCR analysis of the microdissected primary palate in *Cbfb* mutants demonstrated that the expression of *Tgfb3* was not induced (C), but expression of *Mmp13* was significantly upregulated by TGFB3 application (D). pp, primary palate; sp, secondary palate. *P<0.05 (Mann-Whitney *U*-test; data are mean+s.d.). Scale bar: 200 μm.

In our organ culture system, after 48 h of culture the palatal process fused in control explants, but did not fuse in mutant explants (Fig. 4A). TGFB3 beads rescued the mutant cleft by 80% (4/5), whereas bovine serum albumin (BSA) treatment did not rescue it at all (0/6; Fig. 4A,B), indicating that *Tgfb3* is crucial for cleft palate formation in *Cbfb* mutants. Analysis using qPCR demonstrated that the application of TGFB3 protein resulted in upregulation of *Mmp13* expression without *Tgfb3* induction in the microdissected tissue (Fig. 4C,D). Together, these findings indicate that *Tgfb3* is a crucial target in the pathogenesis of the *Cbfb* mutant cleft.

### Stat3 activity in the Cbfb mutant palate

In our previous study using *Runx1* mutant mice, we demonstrated that Stat3 phosphorylation was disrupted by *Runx1* deficiency in the anterior region of the palate (Sarper et al., 2018). We therefore explored whether or not Stat3 activity is affected during anterior palatal fusion in *Cbfb* mutants.

**Fig. 5.**
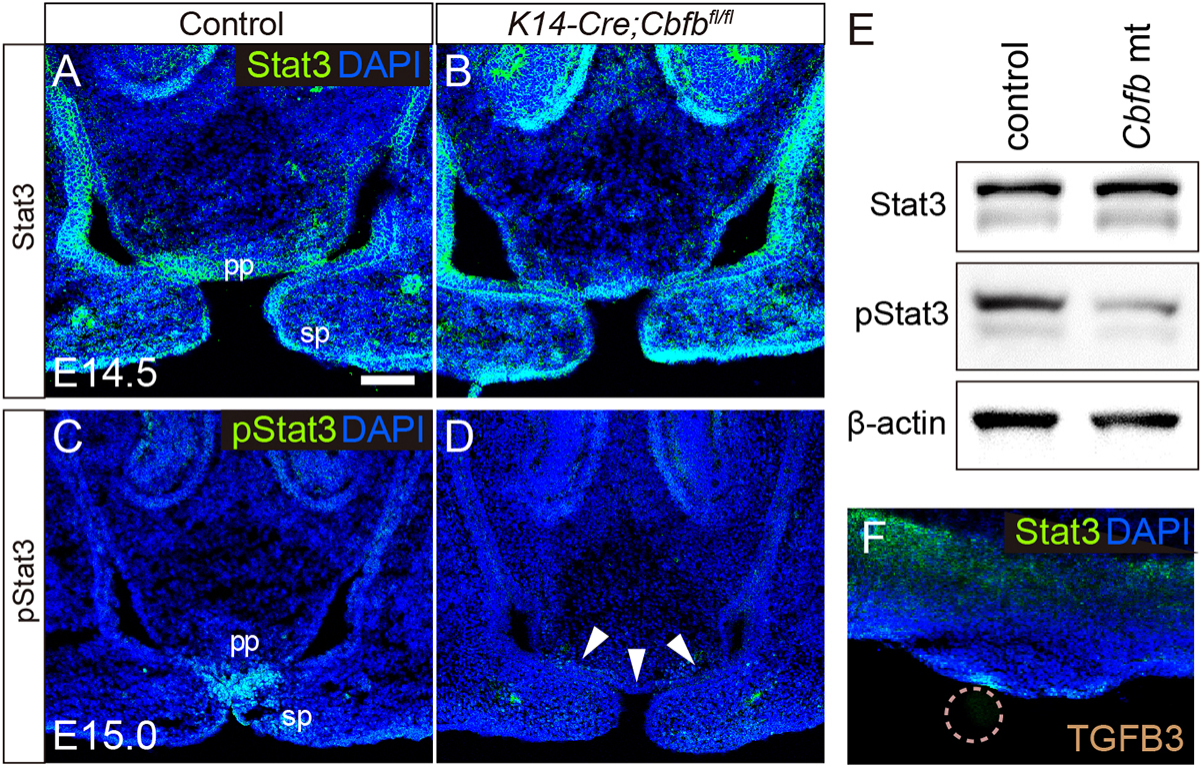
**Stat3 activation in the *Cbfb* mutant palate.** (A-D) Immunofluorescence analysis of Stat3 (green; A,B) and phosphorylated Stat3 (pStat3, green; C,D) in control (A,C) and *Cbfb* mutant mice (B,D). The nuclei were counterstained with DAPI (blue). pStat3 immunoreactivity was downregulated specifically at the anterior region of the palate (arrowheads in D). (E) A western blot confirmed that pStat3 immunoreactivity was specifically downregulated in the primary palate of *Cbfb* mutants. (F) Frontal section of the palatal explants of control mice demonstrated that TGFB3 protein beads ectopically upregulated the pStat3 immunoreactivity. pp, primary palate; sp, secondary palate. Scale bar: 100 μm in A for A-D.

Immunoreactivity to Stat3 was present in the palatal epithelium, and some immunoreactivity was also observed in the mesenchyme (Fig. 5A). *Cbfb* deficiency did not affect the Stat3 immunoreactivity (Fig. 5B). By contrast, immunoreactivity to phosphorylated Stat3 (pStat3) was detected in the fusing or fused epithelium in wild type (Fig. 5C), but pStat3 was remarkably downregulated in the primary palate in *Cbfb* mutants (Fig. 5D). Western blot analysis revealed a significant reduction in immunoreactivity to pStat3 in the *Cbfb* mutant primary palate, although immunoreactivity to Stat3 was not affected (Fig. 5E).

### Stat3 activation by TGFB3

We found that TGFB3 protein ectopically induced Stat3 phosphorylation on the palatal explants (Fig. 5F), suggesting that Stat3 phosphorylation is also regulated by Tgfb3 through some mechanism.

### Rescue of the cleft palate of Cbfb mutants by folic acid

We attempted to rescue the mutant cleft palate using folic acid application. A recent study showed that folic acid and folate activate the Stat3 pathway (Hansen et al., 2015; Wei et al., 2017). We therefore investigated whether or not folic acid application could rescue the anterior cleft palate of *Cbfb* mutants.

**Fig. 6.**
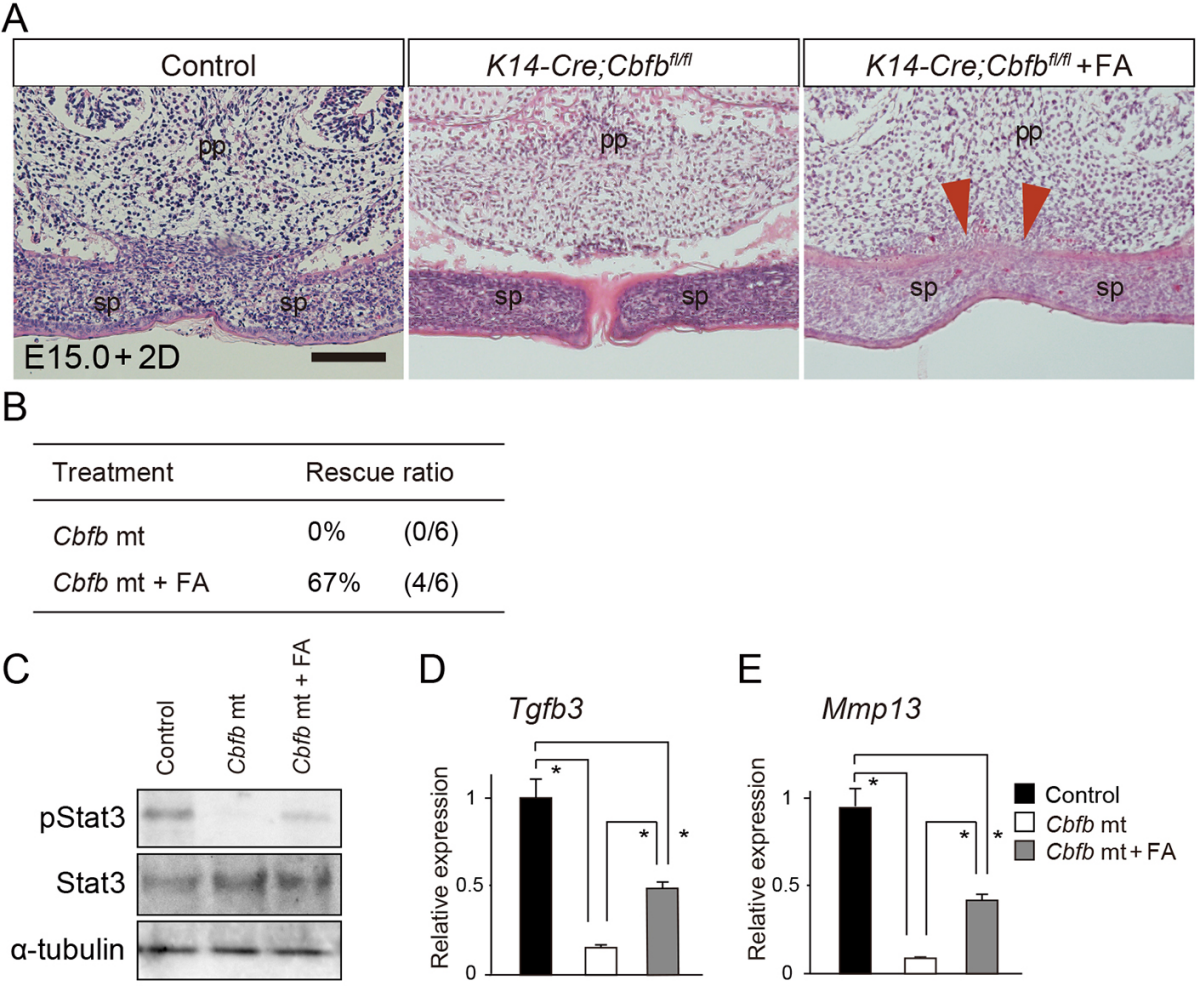
**Folic acid application rescues cleft palate of *Cbfb* mutants.** (A) Histological sections showed that failure of the palatal fusion in *Cbfb* mutants was partially rescued by folic acid (FA) application in culture (arrowheads). (B) The rescue ratio of the cleft palate in *Cbfb* mutants using folic acid. (C) A western blot analysis confirmed that pStat3 immunoreactivity was upregulated by folic acid application in the primary palate of *Cbfb* mutants. (D,E) qPCR demonstrated that folic acid application significantly upregulated expression of *Tgfb3* (D) and *Mmp13* (E) in the palatal tissues of *Cbfb* mutants *in vitro*. pp, primary palate; sp, secondary palate. *P<0.05 (Mann-Whitney *U*-test; data are mean+s.d.). Scale bar: 100 μm.

In our organ culture system, the palatal process fused in control explants after 48 h of culture, but was unfused in the mutant explants (Fig. 6A). Following the application of folic acid for 48 h, histological observation confirmed the partial achievement of mesenchymal continuity by folic acid application in the mutant palatal explants (Fig. 6A), although the mutants did not exhibit fusion of the palatal process between the primary and secondary palates.

Folic acid application rescued the failed palatal fusion with a success rate of 67% (4/6, Fig. 6B). Western blot analysis showed that folic acid application increased pStat3 immunoreactivity, whereas levels of Stat3 were not altered in the dissected mutant primary palate (Fig. 6C). qPCR analysis of the microdissected primary palate revealed that folic acid application upregulated expression of *Tgfb3* and *Mmp13* of the *Cbfb* mutants by almost 50% relative to the control samples (Fig. 6D,E).

## DISCUSSION

Using conditional *Cbfb* null mutant mice, this study provides the first genetic evidence that Cbfb is necessary for palatogenesis. *Cbfb* deficiency resulted in anterior cleft between the primary and secondary palate and led to the failed disintegration of the contacting palatal epithelium, as observed in *Runx1* mutants (Sarper et al., 2018). Cbfb forms a heterodimer with the Runx transcription factors. In hematopoietic development, the functional loss of either *Runx1* or *Cbfb* completely disrupted their function in hematopoietic cells, indicating that Cbfb acts as an obligate cofactor for Runx function (Chen et al., 2011; Chen et al., 2009; Gau et al., 2017). By contrast, *Cbfb* deficiency does not completely disrupt Runx2-dependent bone and cartilage formation (Yoshida et al., 2002), suggesting that Runx2 can regulate skeletogenesis to a limited degree even in the absence of *Cbfb* (Gau et al., 2017); thus, Cbfb seems to act as a dispensable modulator of Runx activity in skeletogenesis (Gau et al., 2017). Given the similarities in the anterior cleft palate observed after the loss of function of *Cbfb* or *Runx1*, Cbfb appears to serve as an obligate cofactor rather than a modulator in Runx1/Cbfb signaling during palatogenesis.

**Fig. 7.**
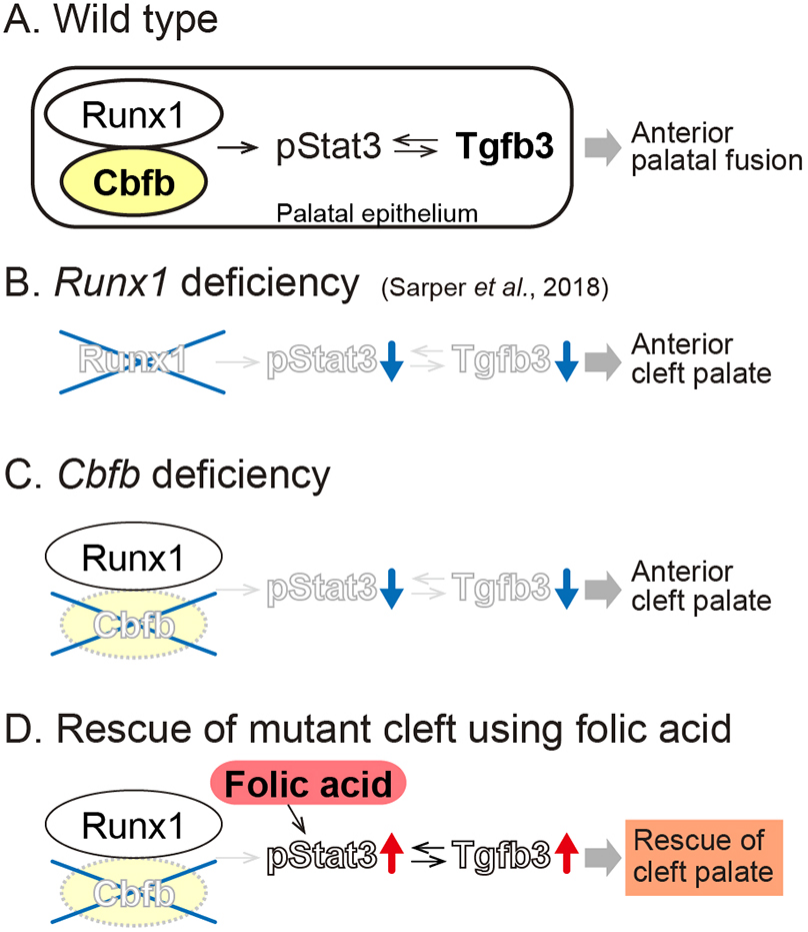
**Schematic of the key findings.** Runx1/Cbfb-Stat3-Tgfb3 signaling regulates the fusion of the anterior palate. (A) In the fusing palatal epithelium of the wild-type palate, Runx1/Cbfb is involved in regulation of Stat3 phosphorylation, which further regulates *Tgfb3* in the anterior region of the palate in a reciprocal manner. (B) *Runx1* mutants exhibit anterior clefting and *Tgfb3* expression is remarkably disrupted in the primary palate, with downregulation of Stat3 phosphorylation, as shown previously (Sarper et al., 2018). (C) *Cbfb* mutant mice also display an anterior cleft palate with downregulation of *Tgfb3* expression and suppressed Stat3 phosphorylation. (D) The anterior cleft palate in *Cbfb* mutants is rescued by pharmaceutical application of folic acid that activates suppressed Stat3 phosphorylation and *Tgfb3* expression.

Our findings provide additional evidence that Runx signaling is important in anterior palatogenesis and that Tgfb3 is a crucial downstream target. As observed in *Runx1* mutants (Sarper et al., 2018), *Tgfb3* expression was specifically downregulated in the *Cbfb* mutants and, conversely, TGFB3 protein beads rescued failed palatal fusion in the mutant. Indeed, epithelial-specific depletion of *Tgfb3*, *Tgfbr1* (*Alk5*) or *Tgfbr2* results in anterior-specific palatal cleft (Dudas et al., 2006; Lane et al., 2015; Xu et al., 2006). Moreover, pharmaceutical Stat3 inhibitor also disrupts anterior palatal fusion with marked downregulation of *Tgfb3* expression (Sarper et al., 2018) and we found that Stat3 phosphorylation was affected in *Cbfb* mutants. Given the obligatory role of Cbfb in Runx1 signaling, the downregulation of *Tgfb3* in the primary palate might account for the anterior-specific clefting in *Cbfb* mutants, as observed in *Runx1* mutants. In addition, these findings provide evidence to support the essential roles of the Runx1/Cbfb-Stat3-Tgfb3 signaling axis in anterior palatogenesis (Fig. 7A-C).

Regarding the mechanisms by which Stat3 phosphorylation is downregulated in *Cbfb* mutants, we previously demonstrated that Socs3, a suppressor of Stat3 phosphorylation, could be involved in the regulation of Stat3 phosphorylation in *Cbfb* mutant mice (Sarper et al., 2018). Socs3 has a binding sequence for the Runx/Cbfb complex and represses its transcription, consequently activating Stat3 phosphorylation through interference with Jak2 (Larsen and Röpke, 2002). In our previous study, *Socs3* was specifically expressed in the primary palate and expression overlapped with the distribution of *Runx1* mRNA. Moreover, this expression was enhanced by *Runx1* deficiency, suggesting that Stat3 phosphorylation could be regulated, at least in part, through the Runx1-Socs3 signaling axis at the anterior palate. By contrast, the promoters of *Tgfb1* and *Tgfb2*, but not *Tgfb3*, contain Runx1 consensus sites within 1 kb of the transcription start site (VanOudenhove et al., 2016). Indeed, the *Tgfb3* promoter does not contain Runx1 consensus sites within 7 kb of the transcription start site, suggesting that some molecules could indirectly mediate the downregulation of *Tgfb3* in *Cbfb* mutants. The present *in vitro* study demonstrated that TGFB3 protein ectopically induced Stat3 phosphorylation on the palatal explants. Although it is still unclear how *Tgfb3* expression is regulated in *Cbfb* mutant mice, our findings, together with those of our previous study, indicate that Stat3 phosphorylation could be reciprocally regulated by Tgfb3 and that Runx/Cbfb signaling might regulate the expression of Socs3, which could further suppress Stat3 phosphorylation.

One of the more striking findings is that the folic acid application rescued the cleft palate in *Cbfb* mutants. In humans, maternal folic acid supplementation has been proven an effective intervention for reducing the risk of non-syndromic cleft palate (Millacura et al., 2017; Wehby and Murray, 2010); however, the mechanism by which folic acid prevents structural anomalies in the fetus is still unknown (Obican et al., 2010). A recent study showed that folic acid can activate Stat3 (Hansen et al., 2015; Wei et al., 2017). In the present study, phosphorylation of Stat3 was activated by folic acid application in the dissected palatal tissue in culture. Conversely, a Stat3 inhibitor impaired anterior palatal fusion between the primary and secondary palates and disrupted the expression of *Tgfb3 in vitro* (Sarper et al., 2018). Taken together, these findings show that folic acid rescued the cleft palate of *Cbfb* mutants, presumably through the activation of Stat3. Furthermore, the rescue of the mutant cleft palate using folic acid might highlight potential therapeutic targets aimed at Stat3 modification for the prevention and pharmaceutical intervention of cleft palate (Fig. 7D).

In conclusion, the present study demonstrated that Cbfb is essential for anterior palatogenesis, acting as an obligatory cofactor of Runx1/Cbfb signaling (Fig. 7A). In addition, we demonstrate the rescue of mutant cleft palate through pharmaceutical folic acid application, at least in part, by activating Stat3 phosphorylation in the Runx/Cbfb-Tgfb3 signaling axis during palatogenesis (Fig. 7D).

## MATERIALS AND METHODS

### Study approval

All of the animal experiments were performed in strict accordance with the guidelines of the Animal Care and Use Committee of the Osaka University Graduate School of Dentistry, Osaka, Japan. The protocol was approved by the Committee on the Ethics of Animal Experiments of Osaka University Graduate School of Dentistry. Mice were housed in the animal facility at the Department of Dentistry, Osaka University. Welfare guidelines and procedures were performed with the approval of the Osaka University Graduate School of Dentistry Animal Committee.

### Animals

*Cbfb*^−/−^ mice are early lethal owing to hemorrhaging between E11.5 and E13.5, when the palatal development is not yet initiated (Sasaki et al., 1996). To assess the role of Cbfb in the oral epithelium, we use epithelial-specific knockout mice created through the *Cre/loxP* system (*K14-Cre/Cbfb^fl/fl^*), as described in a previous study (Kurosaka et al., 2011). We used their littermates that did not carry the *K14-Cre/Cbfb^fl/fl^* genotype as controls.

### Assessment of palatal fusion and histological analysis

The palatal phenotypes were first evaluated with a dissecting microscope. For histology, dissected samples were fixed in 4% paraformaldehyde at 4°C overnight. The samples were then dehydrated, embedded in paraffin, serially sectioned at 7 μm and stained with Hematoxylin and Eosin. For cryosections, the samples were dehydrated in 15% and 30% sucrose in diethyl pyrocarbonate (DEPC)-treated PBS overnight at 4°C and then embedded in Tissue-Tek (OCT compound, Sakura). The tissue samples were sectioned into 10 μm slices.

### Immunohistochemistry

Immunofluorescence staining was performed using polyclonal rabbit-anti-Ki67 (1:400, ab15580, Abcam), polyclonal rabbit anti-K6 (1:200, #4543, 905701, Biolegend), monoclonal anti-K14 (1:200, ab7880, Abcam), monoclonal rabbit anti-phospho-Stat3 (pStat3, 1:200, #9145, Cell Signaling Technology) or monoclonal rabbit anti-Stat3 (1:200, #9139, Cell Signaling Technology) overnight at 4°C. Alexa488-conjugated goat-anti-rabbit IgG (1:400, A21206, Molecular Probes) or Alexa546-conjugated goat-anti-mouse IgG (1:400, A11003, Molecular Probes) was used as secondary antibody. DAPI (1:500, Dojindo) was used for nuclear staining and the sections were mounted with fluorescence mounting medium (Dako). At least three embryos of each genotype were used for each analysis.

The percentage of proliferating cells at the fusing or contacting epithelium between the primary and the secondary palate was determined by counting Ki67-positive cells and reporting this value as a percentage of the total number of cells, as determined by DAPI staining.

### Laser microdissection

The dissected heads were freshly embedded in Tissue-Tek and frozen immediately. Then, tissues were serially sectioned at a thickness of 25 μm using a cryostat (Leica CM 1950). Sections were mounted on a film-coated slide. From the anterior palate at E15.0, 12 to 14 serial sections were obtained in total and stained with Cresyl violet. Palatal tissues at the border between the primary and secondary palate were dissected from the sample sections using a manual laser-capture microdissection system (LMD6500, Leica) and collected into tubes.

### RNA extraction and qPCR analysis

Total RNA was extracted from the laser-microdissected tissues or dissected tissues using IsogenII (Nippon Gene) according to the manufacturer's protocol, then reverse transcribed to cDNA using an oligo (dT) with reverse transcriptase (Takara). For qPCR, the cDNA was amplified using TaqDNA polymerase (Toyobo, Sybr Green Plus) using a light cycler (Roche). *Gapdh* was used as a housekeeping gene. Primer sequences have been reported previously (Sarper et al., 2018). At least three embryos of each genotype were used for each analysis.

### Whole-mount *in situ* hybridization

Whole-mount *in situ* hybridization was performed using fixed E14.0, E14.5 and E15.0 palates. The digoxigenin-labeled RNA probes were prepared using a DIG RNA labeling kit according to the manufacturer's protocol (Roche) employing each cDNA clone as the template. The probes were synthesized from fragments of *Cbfb*, *Runx1*, *Runx2*, *Runx3*, *Shox2*, *Msx1*, *Shh*, *Bmp4*, *Tgfb3* and *Mmp13* (Allen Institute for Brain Science; https://alleninstitute.org/) and amplified with T7 and SP6 adaptor primers through PCR, as described previously (Sarper et al., 2018). After hybridization, the signals were visualized according to their immunoreactivity with anti-digoxigenin alkaline phosphatase-conjugated Fab fragments (Roche). At least three embryos of each genotype were used for each analysis.

### TUNEL staining

To detect apoptotic cells, the TUNEL assay was performed according to the manufacturer's instructions (ApopTag, Chemicon). Frozen sections (10 μm) were prepared and the stained sections counterstained with methyl green. At least three embryos of each genotype were used for each analysis.

The percentage of apoptotic cells along the contacting or fused epithelium between the primary and secondary palate was determined by counting the number of TUNEL-positive cells and reporting this value as a percentage of the total number of cells, as determined by methyl green staining.

### Rescue of the mutant cleft palate using TGFB3 protein or folic acid

The dissected palate of the E15.0 mutants was cultured on a Nuclepore filter (Whatman) in Trowell-type organ culture in chemically defined medium (BGJb, Gibco/Life Technologies). Affi-Gel beads (Bio-Rad) were incubated in TGFB3 (100 ng/μl, R&D Systems) and placed on the primary palate of the *Cbfb* mutant explants, as described previously (Sarper et al., 2018). BSA was used for the control beads. Fusion of the palatal process was evaluated histologically. The anterior portion of the palates was also dissected under the microscope and total RNA was extracted from these samples for analysis by qPCR.

To evaluate the possible rescue of cleft palate in *Cbfb* mutants by folic acid application, the palatal explants were cultured for 48 h in BGJb (Gibco) culture medium containing folic acid (*N*^5^-formyl-5,6,7,8-tetrahydropteroyl-L-glutamic acid, Sigma-Aldrich) at a final concentration of 100 μg/ml. After culture, the *in vitro* explants were fixed at each stage in 4% paraformaldehyde overnight and then processed for histological observation.

### Induction of Stat3 phosphorylation by TGFB3

To evaluate the possible induction of Stat3 phosphorylation by TGFB3 protein, the dissected palate of E15.0 mutants was cultured and TGFB3 (100 ng/μl, R&D Systems) protein beads were placed on the explants, as described above. Ectopic phosphorylation was evaluated by immunohistochemical staining of pStat3 on the frontal section.

### Western blot analysis

For western blotting, the primary palate of *Cbfb* mutants was dissected and cut in half. Each half of the explant was cultured with or without folic acid for 48 h.

The dissected samples were lysed with radioimmunoprecipitation assay (RIPA) buffer (Nacalai Tesque) supplemented with protease and phosphatase inhibitors (Nacalai Tesque). The lysates were centrifuged and the supernatant was heated in denaturing Laemmli buffer (Bio-Rad). Proteins were separated using SDS-PAGE and transferred to polyvinylidene difluoride membranes (Bio-Rad).

The membranes were incubated with anti-Stat3 (1:1000, #9139, Cell Signaling Technology), anti-pStat3 (1:1000, #9145, Cell Signaling Technology), anti-β-actin (1:2000, A1973, Sigma-Aldrich) or anti-α-tubulin (1:1000, T6074, Invitrogen) antibodies. The bound antibodies were detected using horseradish peroxidase (HRP)-linked antibodies (anti-mouse, 1:1000, 172-1011, Bio-Rad; anti-rabbit, 1:1000, 656120, Invitrogen) and an ECL detection kit (Bio-Rad).

### Statistical analyses

Quantitative variables in two groups were compared using the Mann-Whitney *U-*test. Differences among three groups were determined using the analysis of variance (ANOVA) test; significant effects indicated by the ANOVA were further analyzed using post-hoc Bonferroni correction. *P-*values <0.05 were considered significant. Significance was determined using the statistical analysis software program JMP, version 5 (SAS Institute Inc.)

## Supplementary Material

Supplementary information
