# Supplementary material for: Anterior cleft palate due to Cbfb deficiency and its rescue by folic acid
Source: Dis Model Mech. 2019 Jun 27;12(6):dmm038851. doi: 10.1242/dmm.038851 (PMC6602316; doi:10.1242/dmm.038851)
Supplement: Supplementary information [file dmm-12-038851-s1.pdf]

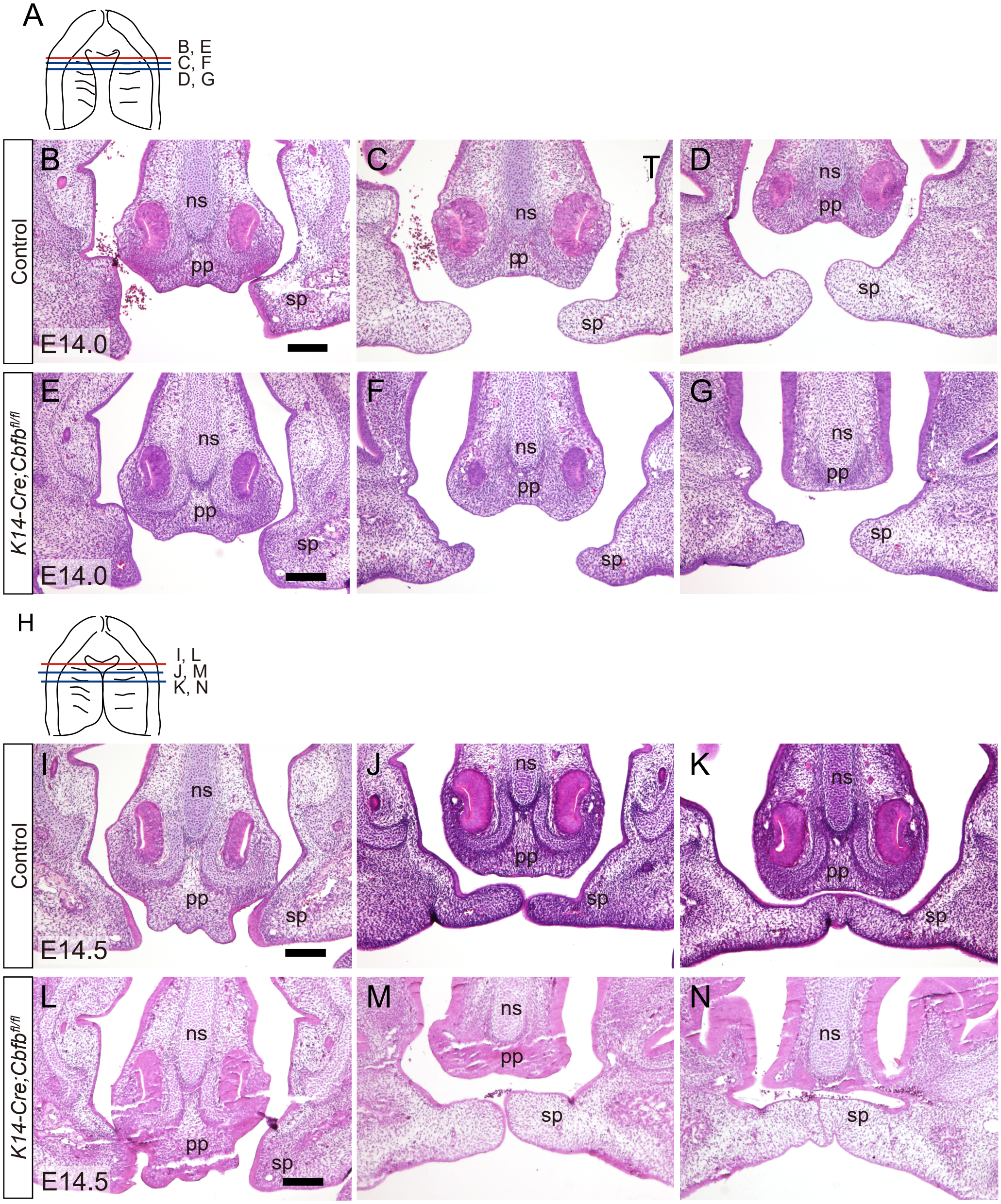

Figure S1. Sequential frontal sections at E14.0 and E14.5.

(A,H) The diagram shows the occlusal view of the palate and the section positions as indicated by the lines. (B-G, I-N) Histological frontal sections at E14.0 (B-G) and E15.0 (I-N) of *Cbfb* mutant and control mice. Palatal phenotypes were not evident in these stages. Scale bar: 200 μm.

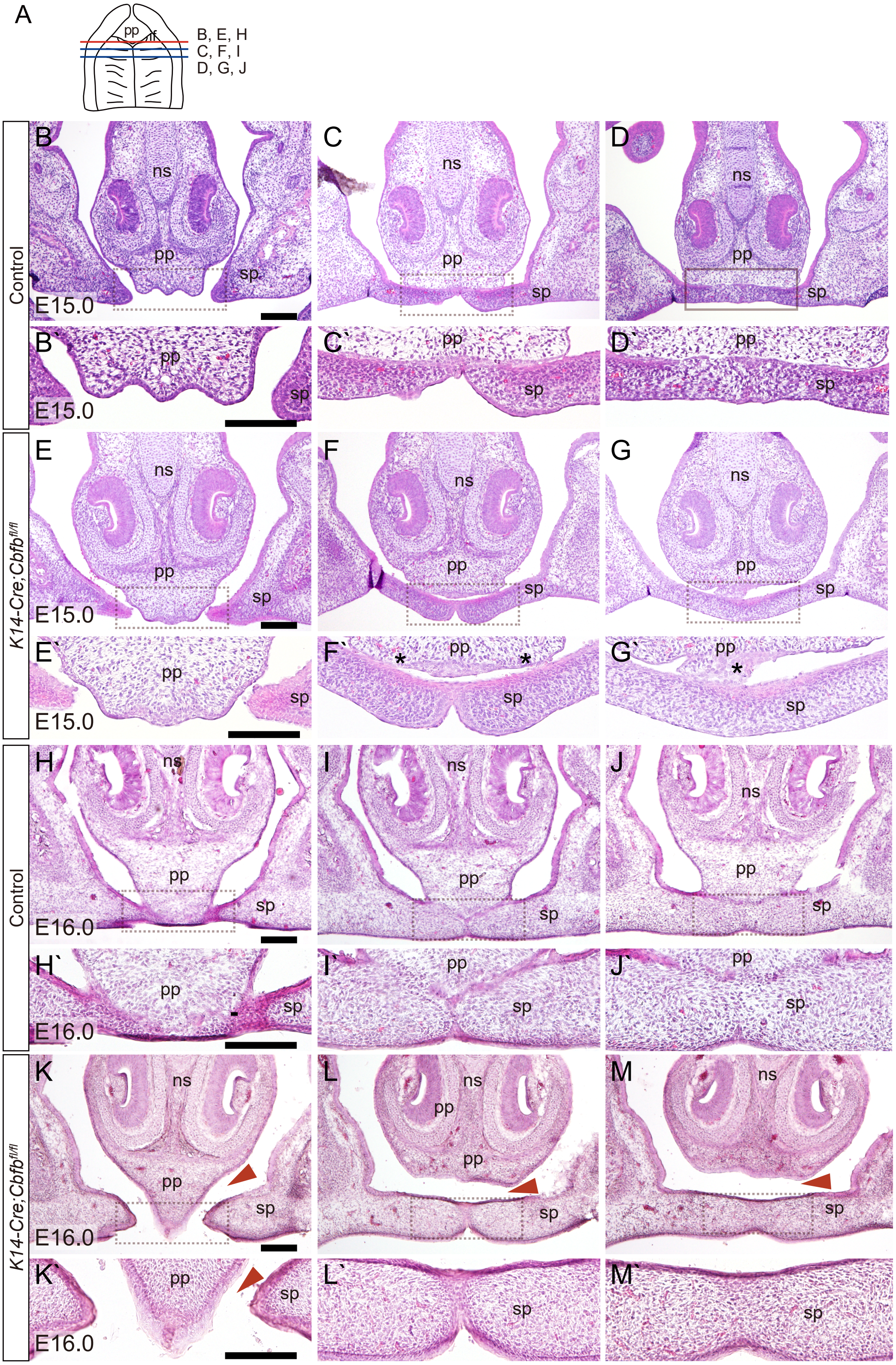

Figure S2. Sequential frontal sections at E15.0 and E16.0.

(A) The diagram shows the occlusal view of the palate and the section positions as indicated by the lines. (B-M) Histological frontal sections at E14.0 (B-G) and E15.0 (H-M) of *Cbfb* mutant and control mice. (B`-M`) Higher magnification views (inset of panel B -M). Arrowheads (K,L,M) indicate the failure of fusion. Asterisks (F`,G`) indicate the contact between the primary and the secondary palates.
